# Quantifying light scattering with single-mode fiber -optic confocal microscopy

**DOI:** 10.1186/1471-2342-9-19

**Published:** 2009-11-19

**Authors:** Jeffrey T LaCroix, Mark A Haidekker

**Affiliations:** 1 University of Missouri, Department of Biological Engineering, Columbia, MO 65211, USA; 2 University of Georgia, Faculty of Engineering, Athens, GA 30602, USA

## Abstract

**Background:**

Confocal microscopy has become an important option for examining tissues *in vivo *as a diagnostic tool and a quality control tool for tissue-engineered constructs. Collagen is one of the primary determinants of biomechanical stability. Since collagen is also the primary scattering element in skin and other soft tissues, we hypothesized that laser-optical imaging methods, particularly confocal scattered-light scanning, would allow us to quantify scattering intensity and determine collagen content in biological layers.

**Methods:**

We built a fully automated confocal scattered-light scanner to examine how light scatters in Intralipid, a common tissue phantom, and three-dimensional collagen gels. Intralipid with 0.5%, 1.0%, 1.5%, and 2.0% concentration was filled between precisely spaced glass coverslips. Collagen gels at collagen concentrations from 0.30 mg/mL to 3.30 mg/mL were prepared, and all samples underwent A-mode scanning with multiple averaged scans. In Intralipid samples, light reflected from the upper fluid-glass interface was measured. In collagen gels, average scattering intensity inside the actual gel was measured. In both cases, intensity was correlated with concentration.

**Results:**

By measuring light attenuation at interface reflections of various thicknesses using our device, we were able to determine that the scattering coefficient at 660 nm of Intralipid at increasing concentrations in water to be 39 cm^-1 ^for each percent increase of Intralipid. We were also able to measure the amount of scattering of various concentrations of collagen in gels directly using backscattered light. The results show a highly linear relationship with an increase of 8.2 arbitrary units in backscattering intensity for every 1 mg increase of collagen within a 1 mL gel volume.

**Conclusion:**

The confocal scattered-light scanner allows to accurately quantify scattering in Intralipid and collagen gels. Furthermore, a linear relationship between collagen concentration and intensity was found. Confocal scattered-light scanning therefore promises to allow imaging of collagen content in soft tissue layers.

## Background

Confocal microscopy is most widely used to image fluorescence - either intrinsic or extrinsic - of an object, such as biological tissue. Different information can be obtained from the tissue under examination when the light scattering properties are examined. The hypothesis of this study is that light scattering quantitatively depends on collagen content and that scattered-light confocal microscopy can be used to determine collagen content in tissues.

Being able to analyze collagen content in tissue is important for several fields of research, including tissue physiology and tissue engineering. Collagen plays a key role in soft tissue repair [1] and is an important determinant in tissue engineered constructs, such as the cornea [2], the heart valve [3], and tissue-engineered blood vessels [4]. Collagen is a major source of scattering in skin *in vivo *[5], and therefore is a main source of contrast in skin imaging. Confocal microscopy is beginning to establish itself as a method of examining tissues *in vivo *as a diagnostic tool for the human cornea [6,7] and for skin [8,9]. This technique has also been examined for *in vitro *quality control of tissue engineered constructs [10,11]. It has been suggested that collagen plays an important role in the maximum load force of tissue-engineered constructs [12-14]. For purposes of tissue engineering, quantification of collagen content may provide a tool to predict biomechanical stability *in vivo*. In spite of the important role that collagen plays in tissue, noninvasive methods to determine collagen content are not readily available.

Collagen is the primary source of light scattering contrast in the visible range of light, and some studies have examined epithelial scattering coefficient with confocal microscopy [15,16]. To verify our hypothesis that collagen content can be recovered from scattering information, we used a two-pronged approach based on a scattered-light confocal system to examine scattering properties of Intralipid and collagen gels. Intralipid is fluid that is commonly used as a phantom with similar optical properties as that of biological tissues [17,18]. The properties of Intralipid have been thoroughly studied, providing us with a reliable standard for comparison [19,20]. The purpose of using Intralipid in this study is to establish a quantitative relationship between actual scattering of the fluid and the measured signal. Collagen gels are typically used as *in vitro *systems used to model cell behavior in three dimensions [21-24]. Collagen gels have also been proposed as skin dressing for wound healing [25], and are a very popular material for scaffolds in tissue engineering, e.g., for vascular grafts [26,27], tissue-engineered tendons [28] or tracheal grafts [29]. The use of collagen gels constitutes the second part of the two-pronged approach in this study where we validate the hypothesis that collagen content is directly related to scattered light in the confocal microscopy system. We found a strictly linear relationship between Intralipid concentration and scattered light intensity and a linear increase of scattered light intensity with collagen content in collagen gels.

## Methods

**Figure 1:**
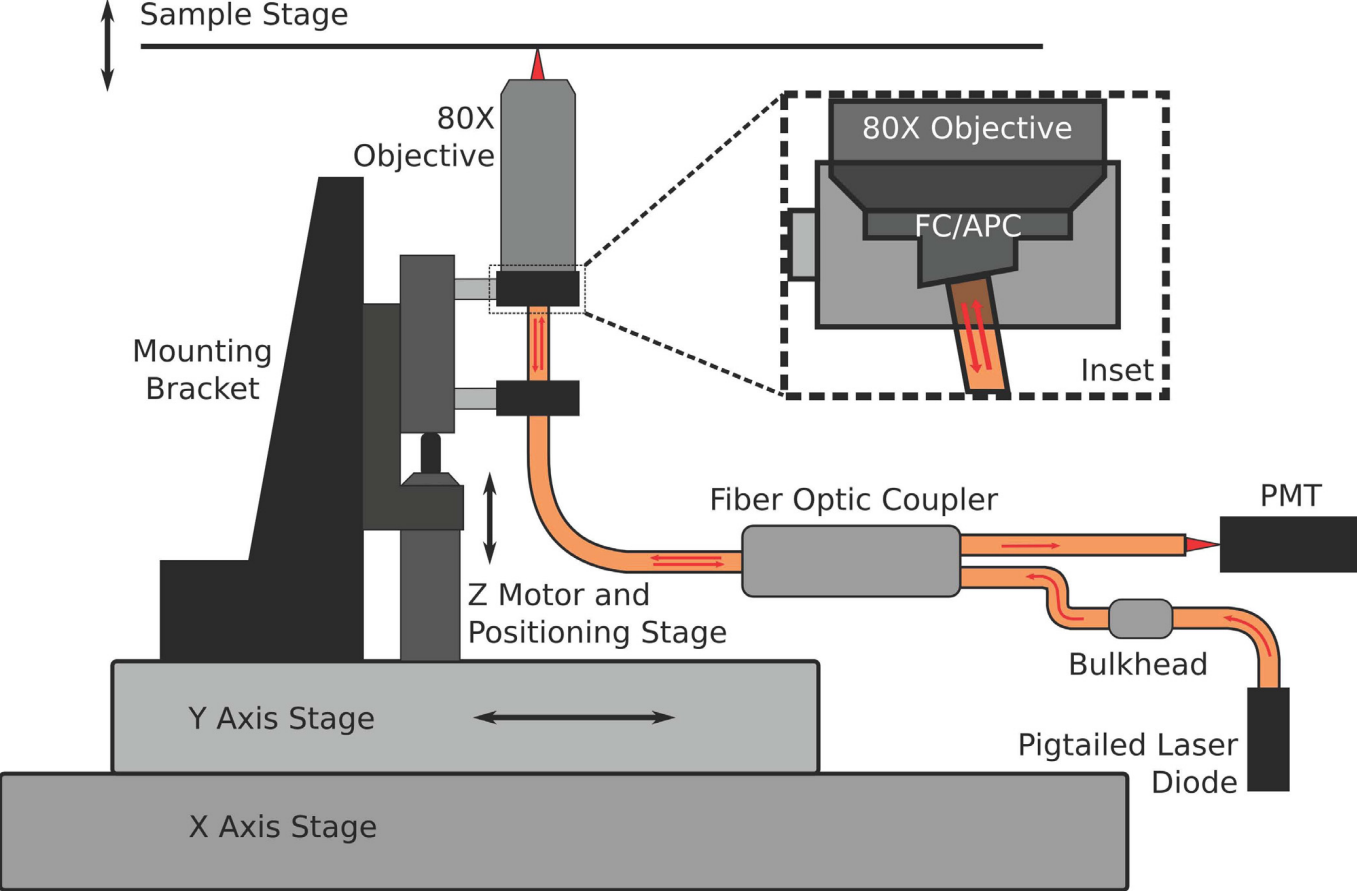
**Schematic of the confocal scanner**. The inset shows the FC/APC (fiberoptic coupling with angle-polished coating) connection used to minimize reflection at the fiber-air interface.

Our scattered-light confocal scanner was based on to the principle of single-pinhole confocal microscopy and shown in Figure 1. The device used in this study is a modified version of a device that was described in detail elsewhere [10]. A single-mode fiber optic coupler (Fiber Optic Network Technology Co, Surrey, British Columbia) was used in place of conventional free-space optics in order to avoid alignment issues. A 7.5 mW, 660 nm laser diode fiber pigtail (Thorlabs, Newton, NJ) was attached to one of the input ports of the fiber optic coupler via bulkhead. The output of the fiber optic coupler served as a small pinhole, illuminating the sample while collecting reflected and back-scattered light from the sample. The output port was fitted with a FC/APC termination and polished at an 8 degree angle in order to avoid specular reflection at the fiber-air interface and aligned for a normal optical axis. The illumination light was focused through a 15 mm working distance 80× industrial microscope objective with numerical aperture of 0.5 (Edmond Optics, Barrington, NJ). Backscattered light was collected by the fiber output port and split between the input fibers, one of which guided the backscattered light to a photomultiplier tube (PMT) (Hamamatsu Photonics, Bridgewater, NJ). This optical system was mounted to a vertical translation stage driven by a DC actuator with built-in encoder which equated one pulse to 163 nm of travel (Thorlabs, Newton, NJ). The scan head was mounted to a customized XY stage driven by stepper motors (Anaheim Automation, Anaheim, CA) for positioning. An additional customized stage was built in order to accommodate the sample and driven vertically by two motorized translational stages (Anaheim Automation, Anaheim, CA).

A single axial scan (A-mode scan) was obtained through the raising of the vertical translation stage towards the sample, changing the focal plane of the objective. Backscattered light from the sample was detected by the PMT after traveling through the fiberoptic coupler. A 10-bit analog to digital converter processed the PMT signal which was displayed as intensity as a function of linear distance. A B-mode scan (multiple A-mode scans along a linear path) could also be obtained using the X-Y positioning capabilities of the instrument.

**Figure 2:**
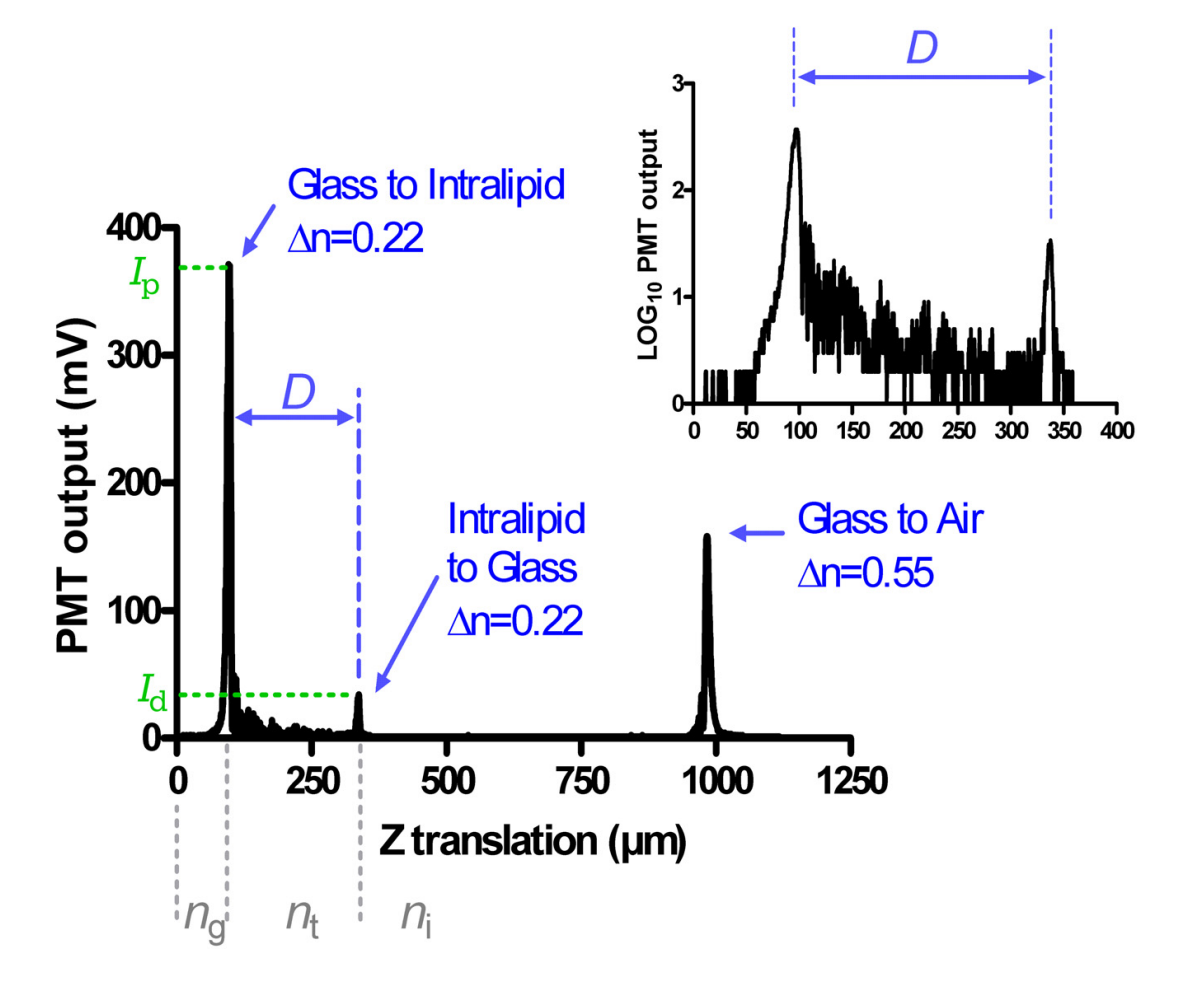
**A-mode scan of Intralipid**. The intensity *I*_d _of the peak resulting from the refractive index change from Intralipid to Glass is recorded and evaluated relative to the first peak *I*_p _that indicates the glass-to-Intralipid interface. Actual thickness *D *is determined by scaling the measured distance between the Intralipid peaks by the refractive index of Intralipid, *n *= 1.33. The inset shows the signal between the glass slides magnified and on a semi-logarithmic scale to better highlight the scattering component.

Sufficient signal-to-background ratio (S/B) is necessary for identification of a source of contrast, such as a reflective interface. S/B is limited as light is focused deep within scattering media since light attenuation is governed by Beer's law. In this first part of the study, the primary source of contrast is refractive index change, caused by reflection between two interfaces. In order to ensure that our signal would not be overly attenuated at the imaging depths relevant to this study, we first computed the maximum depth that we would be able to resolve a refractive index change of Δn = 0.22 (water - glass). The computation was based on Beer's Law under the assumption that attenuation by absorption *μ*_a _is negligible against attenuation by scattering *μ*_s_. The absorption coefficient *μ*_a _in Intralipid at 660 nm is approximately 0.002 cm^-1 ^[20], while the scattering coefficient *μ*_s _varies between 50 and 92 cm^-1 ^[20]. Light attenuation therefore follows Beer's law as given by(1)

where ***I ***is the intensity of light after distance ***t ***from incident light ***I***_***0***_. Since we are concerned with reflected signals at refractive index mismatches, we use Equation 2 to describe specular reflected light(2)

in which ***I***_***ref ***_is the intensity of the reflected light and ***n***_***1 ***_and *n*_***2 ***_are the refractive indices of the two media at the interface. If we consider a four-layer system consisting of air, the lower glass plate of the container holding the media, the scattering media itself, and the top glass layer with variable refractive index, we can predict the maximum imaging depth ***t***_***PD ***_as a function of scattering coefficient and refractive indices of each medium from manipulation of Equations 1 and 2. This yields the following equation(3)

where the limits of detection are given as ratio ***I***_***d***_/***I***_***p ***_(ratio of reflected light from the upper media-to-glass interface relative to the reflected light from the lower glass-to-media interface), and the refractive indices are given as ***n***_***t ***_for the scattering media, ***n***_***g ***_for the lower glass plate of the container, ***n***_***i ***_and for the detecting interface, that is, the upper glass plate. These values are referred to in Figure 2. In the spacial case that ***n***_***i ***_= ***n***_***g***_, Equation 3 simplifies to Equation 4:(4)

**Figure 3:**
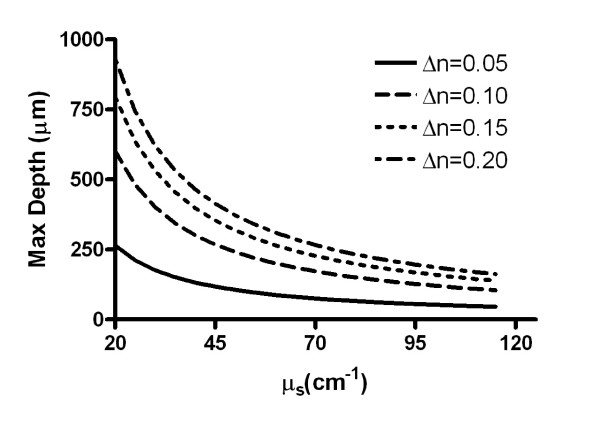
**Theoretical maximum imaging depth due to scattering coefficient at an assumed minimum ratio of 2% for I_d_/I_p _(Equation 3)**. The maximum depth to detect refractive index changes of 0.05, 0.10, 0.15, and 0.20 are shown.

In our calculations, we define maximum imaging depth as the point where ***I***_***d ***_is 2% of ***I***_***p***_. The value of 2% is an estimate, chosen based on our experience with the confocal device so that the intensity would be sufficiently above baseline and noise to be recognized as a peak. Under this assumption, we computed the maximum imaging depth as a function of scattering coefficient of the media at different values for refractive index change Δn (***n***_***t ***_to ***n***_***i***_), with the results shown in Figure 3.

For the first part of this study, 20% Intralipid (Sigma Aldrich) was diluted to concentrations of 0.5%, 1.0%, 1.5%, and 2.0% in ultrapure water. Small volumes of each Intralipid concentration were placed between two glass slides separated by shim spacers of known thicknesses of 127, 191, 254, 318, 381, and 508 *μ*m, creating ay total of 24 samples. Surface tension ensured a homogeneous distribution of the scattering liquid between the closely-spaced glass plates. For each sample, a B-mode scan was taken that consisted of five A-mode scans with a lateral spacing of 3.175 *μ*m. Each A-mode scan had an axial pixel size 326 nm as shown in Figure 2. The five A-mode scans were then averaged for further analysis.

For the second part of the study, we used collagen gels in order to determine the effect of collagen scattering directly, rather than through refractive index changes. Gels were fabricated using high concentration Type 1 (8.00 mg/mL) rat tail collagen (BD Biosciences, Bedford, MA). Twenty-one concentrations were made ranging from 0.30 mg/mL to 3.30 mg/mL at 0.15 mg/mL intervals. Volumes for each desired concentration were mixed into test tubes (Fisher Scientific) which were kept on ice, where volume of collagen was determined as the ratio of the product of desired original volume of solution and final desired concentration to the concentration of the stock volume. 10% of the desired original volume consisted of 10× phosphate buffer solution. 1 N NaOH (2.3 *μ*L per 100 *μ*L collagen) was then added to the test tube. Enough cold 18 MΩ-cm ultrapure water was added to the test tube to bring the solution to a volume of 1 mL. The solution was further diluted with the addition of 333 *μ*L cell culture media per 1 mL original volume and mixed before the addition of the collagen, bringing our total volume to 1.333 mL. 500 *μ*L of each concentration were pipetted twice into 24-well plates (Becton Dickinson Labware, Franklin Lakes, NJ) and allowed to gel for 1 hour at 37°C. One B-mode scan consisting of 30 A-mode scans was taken for each sample where the A-mode scans were spaced 3.175 *μ*m apart and had an axial pixel size 652 nm. The 30 A-mode scans were averaged into one A-mode curve for further analysis. The scattering signal obtained from 196 to 522 *μ*m above the air-glass interface peak was averaged again in order to determine a single-quantity scattering signal. The lower bound was chosen for analysis in order to avoid specular reflection from the well to gel interface while the upper bound was chosen in order to ensure sufficient signal prior to losses due to penetration depth.

## Results

A typical A-mode scan can be seen in Figure 2. In this five-layer system (air - glass - Intralipid -glass - air), four reflection peaks are generated, and the Z scan was adjusted to exclude the first peak, air - glass. Therefore, three reflection peaks are visible where the first two have the intensities ***I***_p _and ***I***_d _as defined in Equation 3. In this sample scan, ***I***_***d ***_is 9.2% of ***I***_*p*_, which is above the detection limit. In between the two peaks, scattered light causes a measurable signal. A comparison of this signal with a scan of a non-scattering medium (background scan) yields a signal-to-background ratio of more than 24 dB. This ratio suggests that the assumed minimum ratio of 2% for ***I***_d_/***I***_p _is a conservative assumption.

**Figure 4:**
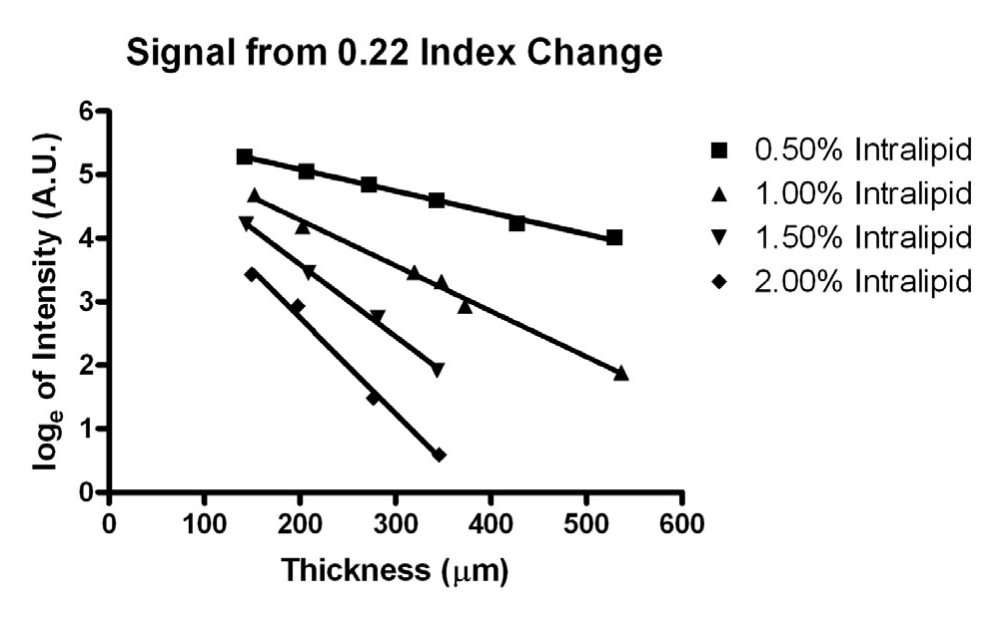
**Natural logarithm of the intensity signal measured from a 0.22 refractive index change (Intralipid to glass) at various thicknesses at four concentrations**. Each concentration was fit to a linear regression to obtain the decay constants.

Taking the natural logarithm on both sides of Beer's Law, Equation 1, leads to Equation 5,(5)

which is the equation of a straight line of the logarithmic intensity ln *I *over the thickness *t*. The magnitude of the slope is the scattering coefficient *μ*_s_. For this reason, the natural logarithms of the intensity of the peak measurements for the Intralipid-glass interface were recorded for each concentration and plotted against thickness of the tissue phantom as shown in Figure 4. Tissue phantom thickness was determined by scaling the peak-to-peak distance that was obtained from the *z *actuator translation by the approximate index of refraction of the Intralipid (assumed to be 1.33) as shown in Figure 2. The natural logarithm of the data where sufficient signal was above baseline was gathered and fit to a linear regression, with the decay constants recorded. For the 0.5% and 1.0% Intralipid concentrations, reflection peaks sufficiently above baseline were recorded for all thicknesses. In the 1.5% and 2.0% Intralipid concentrations, usable peaks were only obtained for the first four thicknesses up to 318 *μ*m. The negative slopes as seen in Figure 4 have values of 3.41 mm^-1^, 7.19 mm^-1^, 11.3 mm^-1 ^and 15.0 mm^-1 ^for the concentrations of 0.5%, 1.0%, 1.5%, and 2.0%, respectively. R^2 ^goodness of fit values were all greater than 0.99 for each concentration. All linear regressions had a significant non-zero slope with P < 0.005. Furthermore, the extrapolation of the lines to *t *= 0 showed that the lines almost converged in one point (5.78 ± 0.055). From Equation 5 it can be seen that this value is the logarithm of *I*_0_, and the low variation between the data sets further confirmed the validity of the measurement.

**Figure 5:**
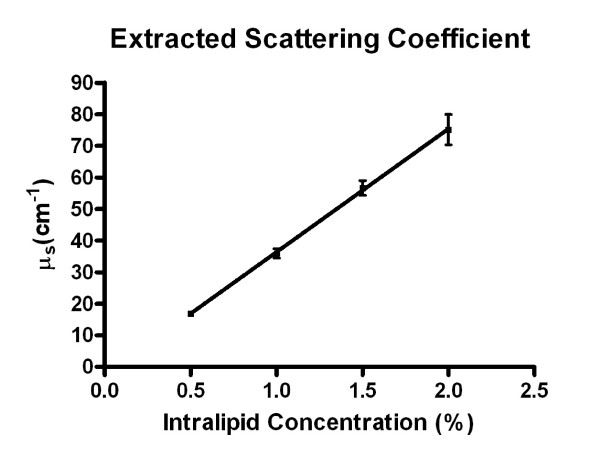
**Extracted scattering coefficient from the decay constants in Figure 4**. Error bars denote 95% confidence of the decay constants. This was fit to a linear regression in order to extrapolate additional concentrations.

**Figure 6:**
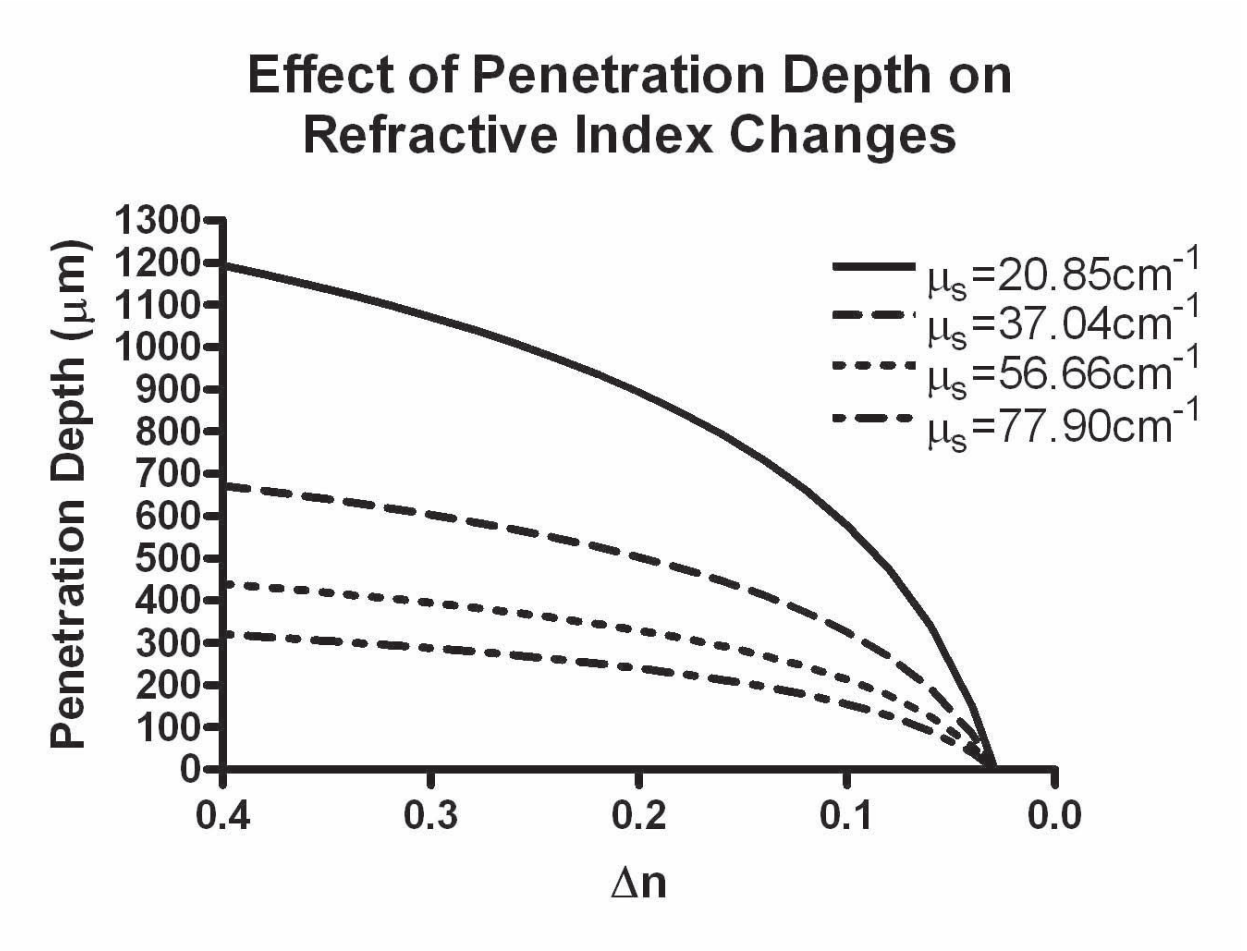
**Theoretical penetration depth assuming minimum ratio of 2% for I_d_/I_p _in order to identify a specific refractive index change Δn at the four scattering coefficients shown in Figure 5**.

The slopes obtained from the regression were used to determine the scattering coefficient of the various concentrations of Intralipid. Considering the round-trip attenuation, the slope values need to be divided by two to obtain the Intralipid scattering coefficient, and we obtained *μ*_s _as 17.0 cm^-1 ^for 0.5% Intralipid, 35.9 cm^-1 ^for 1.0%, 56.7 cm^-1 ^for 1.5%, and 75.1 cm^-1 ^for 2.0%. The values for *μ*_s _were plotted against Intralipid concentration in Figure 5 and showed a clear linear increase of the attenuation coefficient *μ*_s _with Intralipid concentration. Linear regression of the data yielded a slope of 39 ± 0.59 cm^-1 ^per percent. R^2 ^goodness of fit value was approximately 1.0 with P-value 0.0002, showing a significant non-zero slope. Referring back to Equation 3, we were now able to model the maximum imaging depth as a function of refractive index change at the fixed scattering coefficients obtained from Figure 4 in order to validate our peak detections recorded in Figure 4. This model is shown in Figure 6.

**Figure 7:**
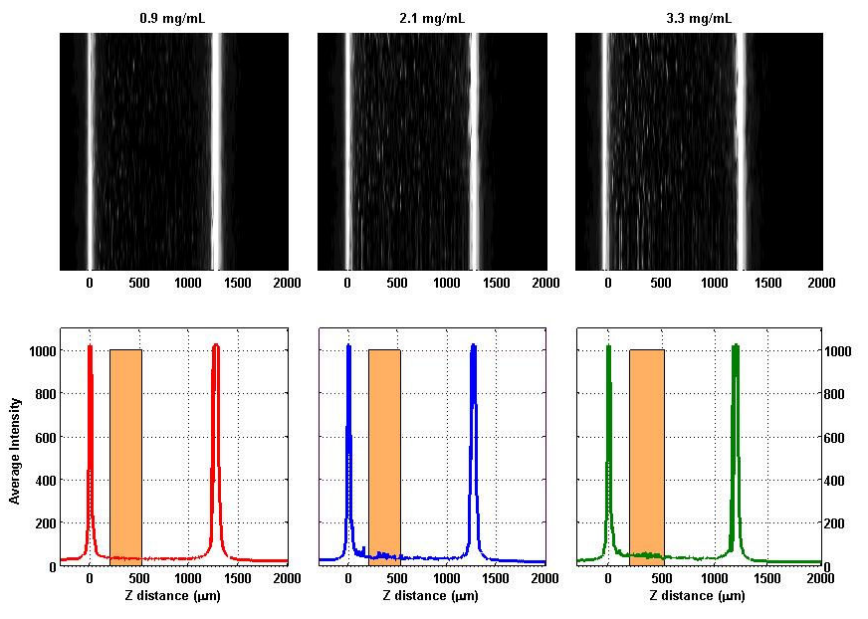
**Selected B-mode scans of collagen gels (top) and their averages (bottom)**. The rectangle indicates the region that was averaged again to determine the single-quantity scattering signal.

**Figure 8:**
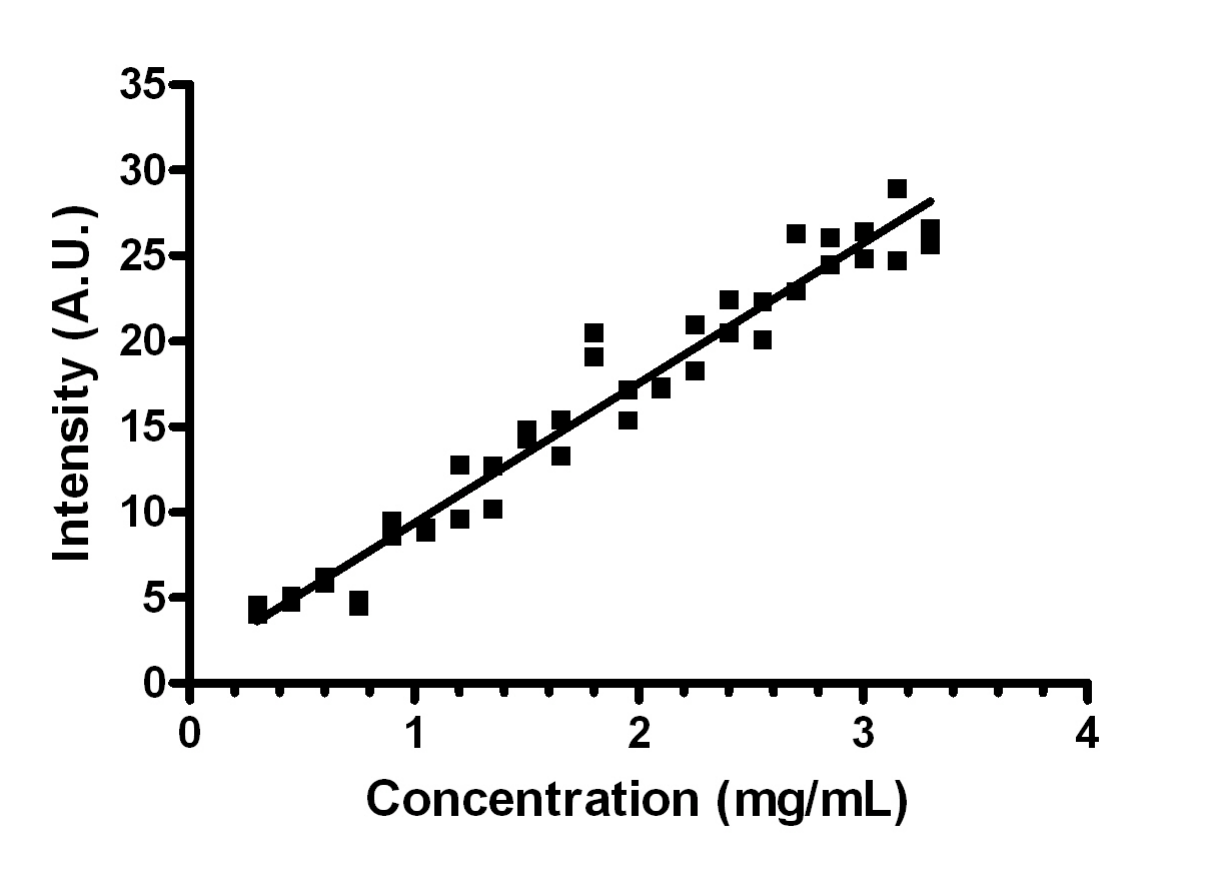
**Quantified backscattering intensity from increasing concentrations of collagen gels**.

In the case of collagen gel scattering, the averaged scattering signal from each of the 42 samples (three of which are shown in Figure 7) was plotted against the collagen gel concentration with the results shown in Figure 8. Linear regression of the data points yielded a slope of 8.2 ± 0.28 (in arbitrary units of intensity per mg/mL). R^2 ^goodness of fit value was 0.95 with P-value of less than 0.0001, showing a significant non-zero slope. Analysis of residuals show a random normal distribution of mean approximately zero and standard deviation of 0.0141. Pearson Product Moment correlation is 2.183 × 10^-9 ^with p-value 1.0, therefore showing no correlation of residuals with the data and indicating our linear model was appropriate in defining collagen scattering.

## Discussion

In this study, we used two different types of samples to examine the ability of the scattering-confocal microscope to quantitatively recover the scattering properties of the samples. First, we attempted to estimate the scattering coefficient of various concentrations of Intralipid by measuring reflection at various depths from refractive index mismatches, and second, we related the absolute scattering signal from collagen gels to collagen concentration.

In strongly scattering media, such as Intralipid, depth imaging ability is important. While a reflection signal can still be attained at high imaging depths, the resolution decreases heavily at higher thicknesses due to specimen-induced spherical aberration [30]. Penetration depth has been examined in confocal microscopy in previous studies [31,32] and is largely governed by confocal pinhole size and absorption in the system. Figure 3 contains an estimate how deep a reflection peak can be detected under the assumption that the second reflection peak (through the media) is at least 2% of the first reflection peak. Under this assumption, we can easily resolve a refractive index change of 0.20 (the approximate refractive index change from an aqueous solution to glass) at thicknesses below 250 *μ*m as long as scattering coefficient is below 70 cm^-1^. The detection limit strongly depends on the signal-to background ratio of the system which we determined to be in the order of 24 dB. This value is predominantly determined by digitization noise, and an analog-to-digital converter with higher resolution could markedly improve the signal-to-background ratio. In practice, however, we found better sensitivity than the theoretical considerations allowed for. From Figure 4, it can be seen that reliable detection of the second peak is possible in 2% Intralipid at 350 *μ*m depth. By using 75 cm^-1 ^for the scattering coefficient, the round-trip transmission through the Intralipid solution is 0.5%, a value that is in excellent agreement with the measured signal-to-background ratio of 24 dB or 1:250. We conclude that the estimate presented in Figure 3 is rather conservative.

From Figure 6 we can verify that our obtained peaks fall within the maximum imaging depths for each shim stock, allowing us to conclude that these peaks were caused by the media-glass interface rather than an unknown artifact or noise. This validates our choice of shim stock thickness for the Intralipid experiments. Within this range of thickness, the intensity of reflected light at the upper interface, the Intralipid-glass interface, is determined by the attenuation of the incident light and the reflected light (round-trip attenuation) by the scattering of the Intralipid. For this reason, we expected the measured intensity of the reflection peak to be dependent on the Intralipid layer thickness following Beer's law for any one concentration of Intralipid. Figure 4 shows this notion to be true. Moreover, the regression slopes allowed us to recover the attenuation coefficient *μ*_s _for the Intralipid dilutions. Figure 5 suggests that *μ*_s _and Intralipid concentrations are linearly dependent for the concentration range examined. For our case, an extrapolation of Figure 5 to 10% Intralipid would yield 390 cm^-1 ^at 660 nm. This value falls between the values measured by Flock [20] and van Staveren [19]. However, scattering is a nonlinear process, and linear extrapolation may not be suitable for higher Intralipid concentrations. Further studies are needed to establish a relationship between scattering coefficient and high Intralipid concentrations. For the purpose of examining engineered tissue sheets, however, the range examined in this study is sufficient. In tissue-engineered blood vessels [33], Gladish et al. determined the scattering coefficient to be 70 cm ^-1 ^[34], which corresponds to an Intralipid concentration of slightly less than 2%. We therefore conclude that the confocal-scattering scanner is capable of accurately determining the scattering coefficient of tissue sheets, provided that the sample can be placed between two thin plates of glass, such as a microscope slice with coverslip. The main difference to the pilot experiments presented in this study is that the availability of a tissue thickness gradient (in analogy to the Intralipid thickness gradient) cannot be assumed. For this reason, a linear least squares fit into intensity/thickness data (Equation 5) is not possible. Rather, the incident intensity *I*_0 _needs to be determined, for example by scanning the glass plates at a location outside of the tissue sample, and *μ*_s _obtained from the average reflected intensity <*I*> at several closely spaced points. Here, Equation 5 would be solved for *μ*_s _with the known intensities <*I*> and *I*_0 _and the thickness *t *as determined from the A-mode scans. Since our confocal scanner has the ability to acquire A-mode scans at different locations, it also has the capability to acquire a spatially resolved scattering map - in other words, an image *μ*_s _(x, y) over the area of the tissue under examination. In applications of tissue engineering, such a scan would provide information about average scattering, homogeneity of scattering, and possible areas of unexpectedly low scattering in the tissue sample. Since collagen is the primary source of scattering in tissue [5], conclusions on collagen expression could be drawn. We anticipate that the confocal technique could be useful in the determination of collagen expression of cells or collagen remodeling of cells that were seeded into a collagen scaffold.

**Figure 9:**
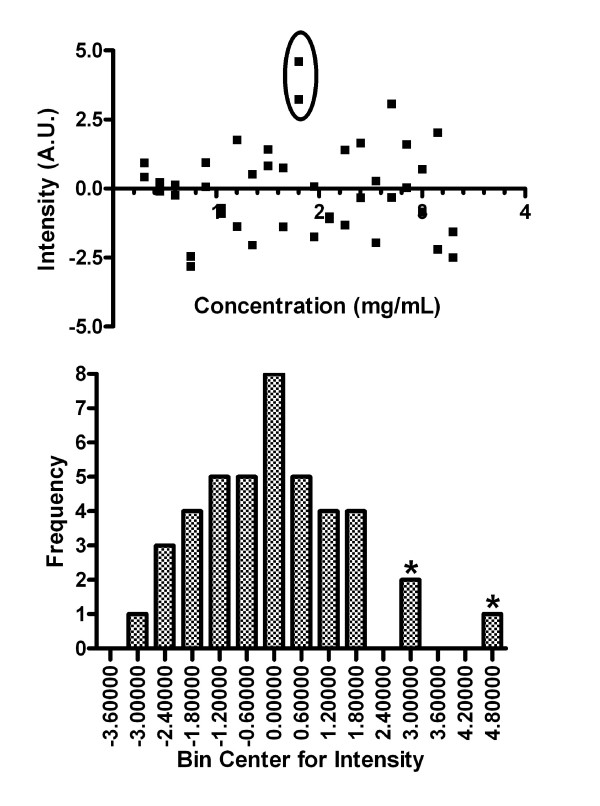
**Residual analysis of Figure 8**. Concentration of 1.8 mg/mL is circled with values denoted as asterisks on histogram.

The main disadvantage of the above method is the invasive nature of the measurement: the tissue needs to be placed between two plates of glass to allow imaging of the scattering map *μ*_s _(x, y). In addition, there is an error of up to 5% in the scaling of the *z*-position of the focal point caused by slight variations in the refractive index of the tissue. For this reason, we investigated the relationship between the scattered light in-between the reflected peaks and collagen concentration in collagen gels. The purpose is to become independent of the second reflection peak as a marker for round-trip attenuation. In such a case, the tissue sheet could be directly examined inside a culture flask without breaking sterility. We found that direct backscattering of light from collagen gels rises as the concentration of collagen within the gels is increasing. In a confocal instrument, backscattering takes place inside the focal volume, that is, inside the collagen gel in our experiments. We found a clear linear relationship between collagen concentration and scattering intensity in the collagen concentration range from 0.3 mg/mL to 3.3 mg/mL. Since the gel was a pure collagen gel, cross-linked collagen was the only scatterer, and we conclude that the increased intensity (Figures 7 and 8) is due to an increase in scattering coefficient of the collagen gel. The statistical analysis supports a linear relationship between scattering intensity and collagen concentration in the examined concentration range. It is worth noting that in the residual analysis, both repeats of collagen gel concentration 1.8 mg/mL are viewed as minor outliers (Figure 9). While it is possible that this is a naturally occurring phenomenon, it is more likely due to a small error in pipetting. Furthermore, the scattering intensity was low for our instrument. Digitization was performed at 10 bits, and Figure 8 clearly shows discretization noise. A stronger light source and higher resolution A/D converter would improve precision.

When the ability to determine the scattering coefficient of a low absorbing medium is combined with the ability to measure backscattering directly, it is possible to generate a calibration curve to relate intensity directly to backscattering for diagnostic imaging purposes. This would provide the possibility to not only determine a relative concentration of collagen in a given area, but to actually quantify the amount of collagen itself. This would particularly be useful in the field of tissue engineering, where collagen scaffolds are necessary to provide tensile stiffness and mechanical strength to the graft [35,36]. Quantifying the amount of collagen within a tissue engineered graft can provide a non-invasive method in predicting biomechanical properties. In further studies of the optical properties of tissue-engineered sheets, it would be interesting to also quantify the scattering anisotropy, because the scattering anisotropy carries information on the assembly of collagen fibrils into larger fiber bundles [37].

## Conclusion

In conclusion, we showed that our confocal scanner can be used to investigate the scattering properties of turbid media. The use of Intralipid demonstrates our ability to measure reflection within low scattering medium in order to extract optical properties when absorption coefficient is negligible. The optical sectioning capability of the single-mode fiber provides the necessary penetration to accomplish this task. The recognition of increased scattering from higher density collagen gels provides supportive evidence that this device can be used to analyze collagen density in terms of contrast for *in vivo *applications.

## Competing interests

The authors declare that they have no competing interests.

## Authors' contributions

JTL and MH jointly designed and built the confocal scattered-light scanner. JTL performed the experiments described in this paper, and MH supervised the study. JTL wrote major parts of the manuscript, and MH contributed most of the introduction and discussion.

## Pre-publication history

The pre-publication history for this paper can be accessed here:

http://www.biomedcentral.com/1471-2342/9/19/prepub
